# Short Lag Times for Invasive Tropical Plants: Evidence from Experimental Plantings in Hawai'i

**DOI:** 10.1371/journal.pone.0004462

**Published:** 2009-02-18

**Authors:** Curtis C. Daehler

**Affiliations:** Department of Botany, University of Hawai'i at Manoa, Honolulu, Hawaii, United States of America; Stanford University, United States of America

## Abstract

**Background:**

The lag time of an invasion is the delay between arrival of an introduced species and its successful spread in a new area. To date, most estimates of lag times for plants have been indirect or anecdotal, and these estimates suggest that plant invasions are often characterized by lag times of 50 years or more. No general estimates are available of lag times for tropical plant invasions. Historical plantings and documentation were used to directly estimate lag times for tropical plant invasions in Hawai'i.

**Methodology/Principal Findings:**

Historical planting records for the Lyon Arboretum dating back to 1920 were examined to identify plants that have since become invasive pests in the Hawaiian Islands. Annual reports describing escape from plantings were then used to determine the lag times between initial plantings and earliest recorded spread of the successful invaders. Among 23 species that eventually became invasive pests, the average lag time between introduction and first evidence of spread was 14 years for woody plants and 5 years for herbaceous plants.

**Conclusions/Significance:**

These direct estimates of lag times are as much as an order of magnitude shorter than previous, indirect estimates, which were mainly based on temperate plants. Tropical invaders may have much shorter lag times than temperate species. A lack of direct and deliberate observations may have also inflated many previous lag time estimates. Although there have been documented cases of long lag times due to delayed arrival of a mutualist or environmental changes over time, this study suggests that most successful invasions are likely to begin shortly after arrival of the plant in a suitable habitat, at least in tropical environments. Short lag times suggest that controlled field trials may be a practical element of risk assessment for plant introductions.

## Introduction

During biological invasions, lag times occur when there is a delay between the time of a species' introduction to a new region and the time when it begins to spread or invade. Lag times could be caused by ecological barriers, such as delayed arrival of a required mutualist [1]. Evolutionary factors, such as time required for recombination and adaptation, have been suggested as potential explanation for lag times [2]. Demographic factors such as Allee effects may also be important [3]. Time lags of more than 50 years between first introduction and subsequent invasion are often assumed to be common in plants [4]. In a statistical analysis estimating lag times for woody plants introduced to Europe, the average time between introduction and first spread (escape from cultivation) was estimated at 170 years for trees and 131 years for shrubs [5]. These lag times were inferred indirectly, based on the time difference between when the plants appeared on planting lists and when they appeared in a published flora or sometimes in unpublished floristic data. The use of heterogeneous historical information presents some limitations: actual dates of introductions are sometimes not known and correspondence between recorded dates of naturalization and actual dates of naturalization or start of spread are typically not known. Furthermore, estimates of lag times are coarse-scale when several decades or more pass in the publication of subsequent floras, and floras vary in comprehensiveness and criteria for including species. An analysis of lag times in Australian cultivated plants using planting lists to indicate date of arrival and herbarium specimens as evidence of naturalization estimated a mode time of 149 years between introduction and naturalization [6]. Analyses of herbarium records [7] can provide valuable information about lag times, but they suffer from limitations because many herbarium specimens are collected opportunistically, potentially long after first naturalization, and the collected plant's status (i.e. planted or naturalized) is not always clear.

The common presumption of long lag times for invasions [8] requires testing through direct and frequent observations of the behavior of many introduced species over time. Wangen and Webster [9] point out that the invasion process can include multiple lag phases. For example, a patchy landscape and a long time to invader maturity can cause sporadic rates of spread long after an invasion has started. This study focuses on the lag time between introduction and the start of spread when plantings have been deliberately made in the immediate vicinity of a semi-natural environment. I used historic planting records from the Harold L. Lyon Arboretum (Honolulu, Hawai'i) and documentation of spread from these plantings to directly determine lag times between planting and the start of spread among 23 plants that subsequently became invasive pests in the Hawaiian Islands.

## Results

**Figure 1:**
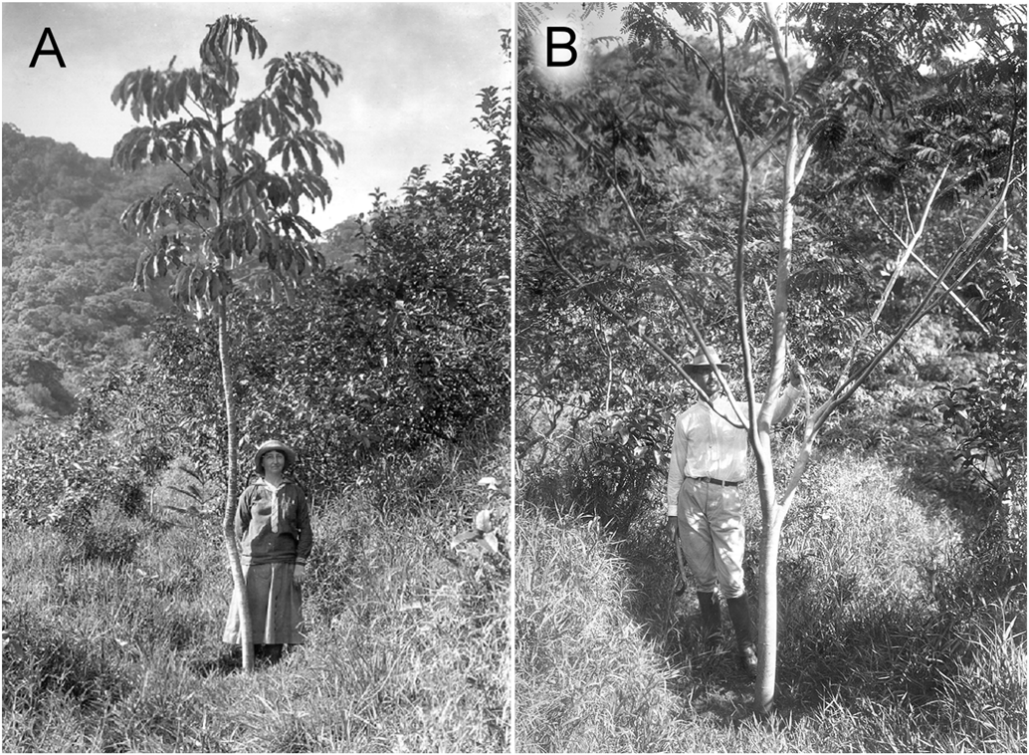
Cecropia obtusifolia (A) and *Falcataria moluccana* (B) are examples of early plantings in the Manoa Valley that became invasive. Natural or semi-natural vegetation is visible in the background of both photos, less than 500 m from these plantings. Photos: E. Caum, 31 December 1922.

**Figure 2:**
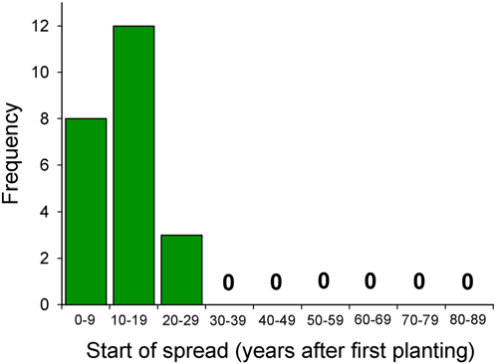
Frequency distribution for time lags between first planting and start of spread in Manoa Valley (Honolulu, Hawai'i) for plant species that became invasive. Records extend for 89 years.

Among hundreds of introduced species that were deliberately planted at the Harold L. Lyon Arboretum, 23 species have become recognized as invasive pests in Hawai'i (Table 1). For these invaders, Lyon's historical records allowed determination of the lag time between planting (Figure 1) and first evidence of spread (Figure 2). Most of the invasive plants (91%) were woody species, with an average lag time of 14 years (range 2–22 years). Among the two herbaceous invaders, the average time lag was 5 years (range 4–6 years). No invaders started to spread between 23 and 89 years after planting (Figure 2). This retrospective analysis does not allow detection of lag times greater than 89 years, but the lack of any invasive species that started to spread in a 66-year period (Figure 2) suggests that most invasions can be expected to start within a decade or two of first planting in disturbed habitats that are in the vicinity of appropriate natural or semi-natural vegetation. Based on field observations (Table 1), the woody invaders require 2–10 years to reach reproductive maturity in Hawai'i, while the herbaceous invaders require 1–3 years; therefore, all of the invaders began to spread within the first few years after reaching maturity.

**Table 1:** Time lag between first planting and start of invasion in Manoa Valley (Honolulu, HI) for plants that eventually became recognized as invasive pests.

Invader	Life form	Year first planted	Year first recorded escaping	Lag (years)	Years from planting to first reproduction 2
*Acacia confusa* Merr.	woody	1922	1938	16	2–4
*Angiopteris evecta* (J.R. Forst.)	herb	1935	1939	4	2–4
*Ardisia elliptica* Thunb.	woody	1920	1938	18	2–3
*Ardisia virens* Kurz^1^	woody	1979	1981	2	2–3
*Cecropia obtusifolia* Bertol.	woody	1921	1932	11	3
*Chrysophyllum oliviforme* L.	woody	1921	1943	22	3–5
*Cinnamomum verum* J. Presl	woody	1921	1939	18	3–5
*Citharexylum caudatum* L.	woody	1931	1937	6	2–3
*Clerodendrum macrostegium* Schauer	woody	1981	1983	2	2–3
*Clusia rosea* Jacq.	woody	1927	1943	16	4–8
*Falcataria moluccana* (Miquel) Barneby & Grimes	woody	1920	1934	14	4–8
*Heliconia latispatha* Benth.	herb	1931	1937	6	2
*Leptospermum polygalifolium* Salisb.	woody	1923	1932	9	2–4
*Macaranga mappa* (L.) Muell.-Arg.	woody	1922	1941	19	2–4
*Macaranga tanarius* (L.) Muell.-Arg.	woody	1922	1939	17	2–4
*Prunus grisea* Kalkman^1^	woody	1985	1989	4	3–4
*Psidium cattleianum* Sabine	woody	1920	1938	18	3–4
*Schefflera actinophylla* (Endl.) H.A.T. Harms	woody	1920	1937	17	3–4
*Spathodea campanulata* Beauv.	woody	1920	1938	18	3–4
*Sphaeropteris cooperi* (Hook. ex F. Muell.) Domin	woody	1927	1934	7	2–3
*Syzygium cumini* (L.) Skeels	woody	1923	1943	20	3–4
*Toona ciliata* Roem.	woody	1921	1941	20	5–10
*Trema orientalis* (L.) Blume	woody	1923	1938	15	4–8

1 So far, recorded only as a pest on the arboretum grounds.

2 Based on personal observations in Hawai'i by the author.

## Discussion

Previously reported lag times [5], [6] were an order of magnitude greater than those reported here. Findings reported here are from a tropical environment, while most prior estimates of time lags have come from temperate environments. It is possible that lag times are shorter in tropical environments where plants can grow year round, and where narrower temperature extremes impose fewer restrictions on establishment. Kowarik [5] noted that many of the species with long lag times in Europe were adapted to warmer climates and their recent spread may have been associated with warming of parts of Europe. In contrast, temperature is unlikely to restrict spread of most tropical invaders. Differences in methodology and circumstances could also explain the shorter lag times reported here. In this study, the initial planting occurred within disturbed but naturally recovering vegetation, in the immediate vicinity of natural or semi-natural environments. In contrast, when date of first introduction is recorded in gardens or urban areas, these locations may be isolated from appropriate natural habitats, hindering spread into natural areas and resulting in long lag times.

A third consideration is that previous estimates of lag times may have been inflated because they were not determined by direct or systematic observations. Naturalization dates determined from herbarium specimens often over-estimate time to naturalization because herbarium specimens are usually not collected in a systematic or regular fashion. Dates of introduction are subject to similar errors, but in some cases they are known more precisely. Analyses of planting lists, floras and herbarium records suggest that the likelihood of detection outside of cultivation, naturalization, and/or area of spread of introduced species increase with residence time [10] and planting frequency [11]. This general pattern is true by necessity, since it is impossible for naturalization or spread to occur instantaneously due to the time required to reach reproductive maturity. But such a pattern is also to be expected due to the nature of herbarium collections: species that have been in residence for a longer period of time, or that have been planted at a greater frequency (more points of naturalization) are more likely to be collected as herbarium specimens. These facts complicate estimates of time lags based on herbarium specimens. It is rare to find studies recording direct field observations of invaders during the early naturalization process, but when such observations have been made, lag times between introductions and the start of spread of an invader have often been short [12]–[14]. Similarly, in the present study, deliberate and frequent searches were made to document spread, and much shorter lag times for plant invasions are evident.

The lag time at the start on an invasion is sometimes defined in mathematical terms as the time required for an invader to reach exponential growth or spread after the first planting or spontaneous occurrence in an area [15]. To determine the lag time using this approach requires analysis of records over many time intervals [15], [16], whereas the present study focused simply on the start of spread for species that became invasive pests. Defining lag time as the transition time to exponential growth is theoretically appealing; however, this lag time is difficult to measure because the exponential growth curve is expected to be exceedingly shallow at the start of exponential growth or spread [17, p. 178], and conclusions are likely to be sensitive to statistical methodologies and assumptions about observation intensity over time. In one of the only experimental studies to examine lag times in this way, Memmott et al. [18] released insect populations of different sizes and deliberately monitored their growth and spread over time. They found that although the smaller populations took longer to reach a given size (an apparent “lag”), the growth of all populations was entirely consistent with exponential growth. There were no unexplained lags. Additional detailed studies of this type are certainly needed. In plants, which commonly require several years to reach reproductive age, heterogeneous rates of exponential expansion are expected at least during the first few generations after arrival [9], so it is important to start with an appropriate null model for exponential growth before seeking alternate explanations for apparent lags.

Although in some cases long lag times are possible due to delayed introduction of a required mutualist [19], change in climate [20], or imposition of a new disturbance regime [21], the results reported here indicate that in all cases, species that became invasive pests began spreading within a few years after the initial plantings reached maturity in disturbed but naturally recovering vegetation. This finding, although from a single tropical region and involving a limited set of species, suggests that carefully controlled field trials [22]–[24] or “in ground” evaluation [25] could become a practical element of risk assessment for plant invaders. In Hawai'i, many invaders could have been identified prior to becoming pests, through their spread within a few years after maturation. A field trial approach is not a substitute for pre-entry risk assessment [26], [27]; rather, it could be most useful and feasible for potentially high-value plant introductions when conventional pre-entry risk assessments are indecisive.

Botanic gardens of all types have great potential to contribute to our understanding of plant invasions, as recently shown by Dawson et al. [28]. Botanical garden records provide a valuable resource documenting planting dates and locations, while observations of spread and plant limitations can be easily made for large numbers of plant species. Closer collaboration between ecologists and botanical garden staff would likely result in new insights into invasion ecology while also yielding new approaches and successes in plant conservation.

## Materials and Methods

### Background and Historic Records

Beginning in 1919, Harold Lyon, working under the auspices of the Hawaiian Sugar Planters Association (HSPA), directed the experimental planting of hundreds of introduced species in the Manoa Valley, near Honolulu, Hawai'i [29]. The planting area consisted of 111 hectares of degraded tropical or sub-tropical wet forest and former sugar cane fields. Lyon's objective was to identify species that could be used for reforestation, allowing restoration of degraded watersheds. He procured plant materials from around the world. Typically, 6–30 individuals of each species were planted as rows, blocks or as scattered individuals. Weeds were initially cleared from around the plantings but persistent clearing or cultivation was not practiced due to limited availability of labor, and perhaps because Lyon wanted to see how these plants would grow on their own. Subsequently, Lyon monitored the plantings and searched for evidence of their spread across the valley. He was particularly interested in identifying species that showed evidence of naturalization. From 1921 to 1950, Lyon and grounds manager Ed Caum produced annual reports to the HSPA detailing their findings and reporting annually on species that were escaping from plantings across the valley. Shortly thereafter, the site came under the management of the University of Hawai'i and eventually it was formally named the Harold L. Lyon Arboretum [29]. Following Lyon's observations, arboretum staff continued to make observations on naturalizing species, recording many of these observations in a database that is used to manage the arboretum grounds. In 2006, Daehler and Baker [30] made systematic surveys in order to identify additional naturalizing species. Only species that were deliberately introduced to the Manoa Valley by Lyon or by subsequent Lyon Arboretum staff were used for analyses because the precise dates of first planting for these species are known.

### Invasive Pest Species

Invasive pest species were independently defined as species meeting at least one of the following two criteria, 1) species that have been listed as significant invaders in Hawaii based on information collected by Smith [31] or 2) species that have become troublesome weeds in the management of the Lyon Arboretum, as documented by efforts made by the staff to control these species [30]. Both of these criteria consider spread and perceived or actual negative impacts in defining invaders.

### Lag Times

Among the species that were deliberately planted at the Lyon Arboretum and that subsequently where classified as invasive pests, I used Lyon's annual reports to HSPA along with notes recorded in the Lyon Arboretum database to determine the time between first planting and first spread in Manoa Valley, as evidenced by naturalized seedlings. Immature plantings are incapable of producing seeds, and this will impose a minimum lag time on each species. To estimate this minimum time, I have reported personal observations on the time required for these species to reach first reproduction in Hawai'i.
